# Consistent prediction of GO protein localization

**DOI:** 10.1038/s41598-018-26041-z

**Published:** 2018-05-17

**Authors:** Flavio E. Spetale, Debora Arce, Flavia Krsticevic, Pilar Bulacio, Elizabeth Tapia

**Affiliations:** 1 Cifasis-Conicet, Santa Fe, Rosario, S2000EZP Argentina; 2 Fceia-UNR, Santa Fe, Rosario, S2000BTP Argentina; 3 Facultad Regional San Nicolás-UTN, Buenos Aires, San Nicolás 2900LWH Argentina; 4 IICAR-Conicet, Santa Fe, Zavalla S2123ZAA Argentina

## Abstract

The GO-Cellular Component (GO-CC) ontology provides a controlled vocabulary for the consistent description of the subcellular compartments or macromolecular complexes where proteins may act. Current machine learning-based methods used for the automated GO-CC annotation of proteins suffer from the inconsistency of individual GO-CC term predictions. Here, we present FGGA-CC^+^, a class of hierarchical graph-based classifiers for the consistent GO-CC annotation of protein coding genes at the subcellular compartment or macromolecular complex levels. Aiming to boost the accuracy of GO-CC predictions, we make use of the protein localization knowledge in the GO-Biological Process (GO-BP) annotations to boost the accuracy of GO-CC prediction. As a result, FGGA-CC^+^ classifiers are built from annotation data in both the GO-CC and GO-BP ontologies. Due to their graph-based design, FGGA-CC^+^ classifiers are fully interpretable and their predictions amenable to expert analysis. Promising results on protein annotation data from five model organisms were obtained. Additionally, successful validation results in the annotation of a challenging subset of tandem duplicated genes in the tomato non-model organism were accomplished. Overall, these results suggest that FGGA-CC^+^ classifiers can indeed be useful for satisfying the huge demand of GO-CC annotation arising from ubiquitous high throughout sequencing and proteomic projects.

## Introduction

Eukaryotic cells are organized into a complex structure of subcellular compartments called organelles. Proteins synthesized in ribosomes can be trafficked to different organelles for the accomplishment of specific physiological functions. Hence, it is not surprising that unexpected protein subcellular localization often underlies the pathogenesis of many human diseases^1–3^. Proteins synthesized in ribosomes can also interact to form macromolecular complexes^4^ -naturally occurring machines inside cells- playing crucial roles in a variety of cellular processes^5,6^. The GO-CC ontology provides a controlled vocabulary for consistent description of both the subcellular structure or macromolecular complex location where proteins may act. Diverse experimental methods can be used to accurately determine the subcellular localization of proteins^7^, ranging from the identification of specific signals on cargo proteins^8,9^ to the use of advanced imaging techniques for revealing protein composition of organelles^10–12^. Similarly, a combination of chemical crosslinking^13^, mass spectrometry, and cryo-electron microscopy^14^ methods can be used to accurately determine the structure and function of macromolecular complexes. Although all these advanced experimental methods are beginning to bear fruits^15,16^, their time-consuming nature and elevated costs^17,18^ make then incompatible with current GO-CC protein annotation demands from ubiquitous large-scale sequencing and proteomic projects. In this scenario, in-silico methods for the automated GO-CC annotation of proteins, i.e., for predicting their localization, at the subcellular structure or macromolecular complex levels, become promising alternatives^19–22^. However, few studies have considered this problem as a whole, CELLO2GO^23^ and FFPred3^24^ being two important exceptions. The CELLO2GO method entails a sequence-based approach for predicting the GO localization of proteins based on their homology to previously localized proteins, mostly belonging to model organisms. On the other hand, the FFPred3 method entails a machine learning-based approach for (separately) predicting all GO domains, including GO-CC, with main focus on divergent human protein chains for which homology-based methods can provide little aid. Since machine learning-based GO annotation methods can overcome the limitations of straightforward homology-based alternatives, they are particularly attractive for the annotation of proteins from non-model organisms.

Taking into account that the study of non-model organisms provides new opportunities for understanding the evolution of multicellular life and cell biological processes^25^, and that substantial reductions in the cost of DNA sequencing have recently burst their study, more efforts on the improvement of machine learning-based methods for GO protein localization are required. In this regard, it is worthy of note that only simplified versions of this problem have been mostly considered in literature. In particular, the prediction of a reduced set of subcellular localization (SCL) categories, often extracted from the SCL section of UniProt entries^26^, has been a frequently revisited problem considering single^27–29^ or multi-category^30–33^ prediction outputs. From a biological point of view, multi-category prediction methods are preferable since relevant proteins often show a ubiquitous character. In either case, protein SCL categories are predicted using the knowledge available at previously localized proteins by characterizing their sequences in terms of a fixed number of informative features. These features may range from the frequency of amino-acids to the existence of low-complexity regions, signal peptides, or trans-membrane helices^34^, among others. On the other hand, the unified prediction of highly-specific protein localization categories derived from *ad-hoc* ontologies like GO-CC has been occasionally considered.

Recalling that an ontology embodies a controlled vocabulary of terms and well-known relationships between them, in-silico methods for GO-CC protein localization can be further differentiated by the consistency of individual GO-CC term predictions. We note, however, that although admittedly important^35^, the consistency problem of ontology-based predictions has been rarely considered in literature. For example, GO-CC FFPred3 predictions are built from a predefined flat -unware of ontology relationships- set of 89 binary GO-CC term predictions; a final propagation step from selected leaf GO-CC terms to the root is then used to accomplish consistent GO-CC predictions. We note, however that consistent GO-CC predictions obtained this way may not be unique, and may not be optimal with respect to the minimization of the probability of erroneous GO-term predictions, since neither the prediction noise of flat GO-CC classifiers, nor the relationships between GO-CC terms are considered. In particular, false positive predictions will be always propagated to the root instead of attempting the prediction of less specific but easier terms, that could improve overall prediction accuracy.

In this paper, a graphical model-based machine learning approach for the automated and consistent GO-CC annotation of protein coding genes is presented. While the graphical component is used to specify the GO-CC ontology, the machine learning component is used to independently learn target GO-CC categories; both these components are then appropriately combined to infer consistent GO-CC annotations. Graphical models have been long used to provide intuitive visions and useful insights in a variety of biological problems at different levels of complexity, including the prediction of metabolic pathways^36^, the prediction of protein functions^37^, and the analysis of complicated drug metabolic systems^38^. Regarding our GO-CC annotation problem, we specifically rely on the power of factor graph models^39^ for obtaining a graphical and formal specification of the GO-CC ontology, for modeling the prediction noise of flat binary classifiers used to predict individual GO-CC categories, for graphically approximating A Posteriori Probabilities (APP) of individual GO-CC categories, for computing corresponding Maximum A Posteriori (MAP) estimates, and for downstream expert analysis of GO-CC predictions. Building upon these concepts, we present FGGA-CC^+^ classifiers, hierarchical ensembles of binary classifiers allowing the straightforward inference of consistent GO-CC annotations by means of the execution of the well-known Sum-Product algorithm^39^ in factor graphs.

Initial insights about FGGA-CC^+^ classifiers were obtained with the former introduction of FGGA classifiers^40^, hierarchical ensembles of binary classifiers defined over GO Molecular Function (GO-MF) compliant factor graphs designed to tackle the automated and consistent GO-MF annotation of protein coding genes. Aiming to deal with GO-CC annotations, FGGA classifiers are now extended into FGGA-CC^+^ counterparts. For this purpose, the factor graph modeling of the transitive *is_a* and *part_of* relationships between GO-CC terms is first considered. In addition, the factor graph modeling of the non-transitive *occurs_in* relationship between GO-CC and GO-BP terms, useful for specifying the subcellular location where a biological process occurs, is complimentary considered. As a result of the *occurs_in* modeling, relevant protein subcellular localization knowledge already available at the GO-BP subdomain can be formally exploited for the enrichment of GO-CC predictions.

## Results

### Characterization of protein sequences for their consistent GO-CC annotation with FGGA-CC^+^ classifiers

Automated GO-CC annotation of protein sequences with FGGA-CC^+^ classifiers requires their characterization in terms of a fixed number of informative protein features. Table 1 shows the average hierarchical Precision (HP), Recall (HR) and F-score (HF) results accomplished by *native* FGGA-CC classifiers, i.e., FGGA-CC^+^ classifiers without the GO-BP enrichment stage, on *A. thaliana* protein sequences. Firstly, a significant effect of characterization methods on hierarchical F-score results is revealed (p < 0.01; Friedman’s test). Secondly, significant differences (see Supplementary Tables S1 and S2) in favor of the Physicochemical^+^ characterization method are observed (p < 0.01; Wilcoxon test with Bonferroni correction).

**Table 1:** Average hierarchical precision (HP), recall (HR) and F-score (HF) accomplished by *native* FGGA-CC classifiers when considering four characterization methods, Signal^+^, Signal^+ +^, PrositeBin, and Physicochemical^+^, on *A. thaliana* protein sequences.

Characterization	HP	HR	HF
PrositeBin	0.78	0.69	0.70
Signal^+^	0.73	0.74	0.71
Signal^+ +^	0.78	0.68	0.70
Physicochemical^+^	0.73	0.79	**0.73**

The best characterization method according to the HF measure (*p* < 0.01; Wilcoxon test with Bonferroni correction) is shown in bold.

### GO-CC annotation of protein sequences with FGGA-CC^+^ classifiers

**Figure 1:**
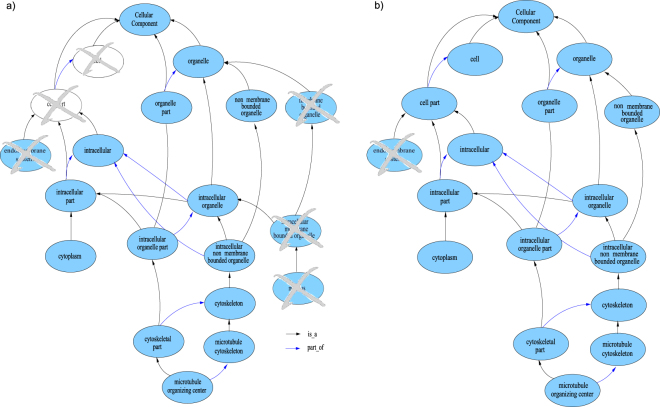
GO-CC subgraphs induced in the annotation of the Q7ZVT3 protein in the *D. rario* model organism. Positive annotations are shown in light blue, negative ones in white, and erroneous ones with a crossline. (**a**) GO-CC annotations accomplished by a naive ensemble of SVM classifiers; erroneous/inconsistent annotations can be observed. (**b**) GO-CC annotations after FGGA-CC^+^ processing; consistent annotations, including just one false positive, can be observed.

FGGA-CC^+^ classifiers were evaluated on protein sequences from five model organisms, *D. rario*, *A. thaliana*, *S. cerevisiae*, *D. melanogaster* and *M. musculus*, using a 5-fold cross-validation approach. In all cases, a Physicochemical^+^ characterization of protein sequences was used, and hierarchical Precision, Recall and F-score performance metrics were evaluated. A first insight into the benefits of requiring consistent GO-CC predictions can be appreciated in Fig. 1 where FGGA-CC^+^ processing over flat GO-CC predictions promotes consistency and reduces the number of false-positives.

**Figure 2:**
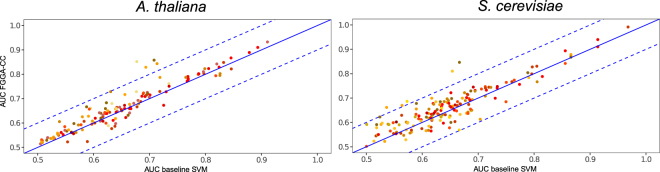
Scatter plots of the average AUC scores attained by *native* FGGA-CC and baseline ensembles of SVM classifiers when performing the GO-CC annotation of protein sequences characterized by the Physicochemical^+^ method. As deeper GO-CC categories are considered, points in the scatter plot turn from yellow to red.

A first round of evaluations was performed to evaluate the baseline annotation performance of FGGA-CC^+^ classifiers. For this purpose, *native* FGGA-CC classifiers were evaluated against naive ensembles of binary SVM classifiers trained to predict just individual GO-CC categories. FGGA-CC classifiers not only yielded better Area Under Curve (AUC) scores but did a particular good job at predicting more specific/deeper GO-CC terms (see Fig. 2 and Figure Supplementary S1).

**Figure 3:**
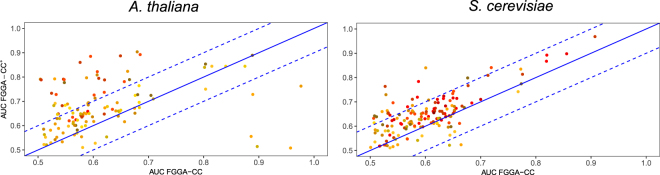
Scatter plots of the average AUC scores attained by GO-BP enriched FGGA-CC^+^ and *native* FGGA-CC classifiers when performing the GO-CC annotation of protein sequences characterized by the Physicochemical^+^ method. As deeper GO-CC categories are considered, points in the scatter plot turn from yellow to red.

A second round of evaluations was performed to evaluate the actual benefits of introducing of SCL knowledge available in boundary GO-BP terms. For this purpose, GO-BP enriched FGGA-CC^+^ classifiers were evaluated against their FGGA-CC alternatives. As expected, FGGA-CC^+^ classifiers yielded higher AUC scores and did a particular good job at predicting even more specific/deeper GO-CC terms (see Fig. 3 and Figure Supplementary S2). Noteworthy, FGGA-CC^+^ classifiers noticeable increased the number of true positive GO-CC annotations (see Figure Supplementary S3). Furthermore, a graphical comparison of predicted GO-CC categories by the two classifiers revealed that these improvements came from positive FGGA-CC^+^ annotations to rather specific GO-CC nodes directly connected, or in the vicinity of, contributing boundary GO-BP nodes. This result makes sense since GO-BP contributing nodes are generally connected to GO-CC nodes located at certain depth. In addition, aiming to quantify observed differences between FGGA-CC^+^ and FGGA-CC classifiers taking into account consistency requirements, the hierarchical precision, recall and F-score performance metrics were evaluated. As expected, the advantages of FGGA-CC^+^ classifiers with respect to FGGA-CC alternatives were confirmed in all but the *D. rario* dataset (see Table 2), for which only one additional boundary GO-BP term was available.

**Table 2:** Annotation performance of *native* FGGA-CC and GO-BP enriched FGGA-CC^+^ classifiers when predicting GO-CC terms for protein sequences in five model organisms.

Organism	HP		HR		HF	
	FGGA-CC	FGGA-CC^+^	FGGA-CC	FGGA-CC^+^	FGGA-CC	FGGA-CC^+^
*D. rario*	72.877	72.914	71.848	72.348	68.313	68.507
*A. thaliana*	73.587	**75.919**	68.471	**76.442**	69.616	**71.190**
*S. cerevisiae*	66.473	**67.248**	83.426	83.896	70.931	**71.935**
*D. melanogaster*	70.690	**72.015**	73.563	74.635	69.531	**71.067**
*M. musculus*	66.592	67.043	77.840	**79.002**	69.952	**70.943**

Protein sequences are characterized with the Physicochemical^+^ method. The average 5-fold hierarchical precision (HP), recall (HR) and F-score (HF) measures are reported. For each model organism, the best performing method according to the HP, HR and HF measures (*p* < 0.01; Wilcoxon test) is shown in bold.

**Figure 4:**
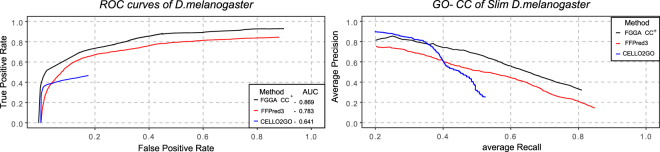
FGGA-CC^+^ (black), CELLO2GO (red) and FFPred3 (blue) GO-CC annotation performance on protein sequences from the *Slim D. melanogaster* dataset. AUC measures favor (*p* < 0.01; Wilcoxon test with Bonferroni correction) the FGGA-CC^+^ method.

For the sake of completeness, a third round of evaluations was performed to evaluate the performance of FGGA-CC^+^ classifiers against two established methods for the automated GO-CC annotation of protein sequences, CELLO2GO and FFPred3. For this purpose, precision, recall and F-score performance metrics and corresponding hierarchical extensions were computed. The CELLO2GO method searches GO-CC annotated homologous proteins in the UniProtKB/TrEMBL database using the Blast algorithm. On the other hand, the FFPred3 method performs an extensive characterization of protein sequences and complementary feature selection before training a naive ensemble of SVM classifiers set to predict an empirically predefined set of 89 GO-CC categories. As a result, comparisons between methods were limited to the 89 predefined FFPred3 GO-CC categories and to the *D. melanogaster* model organism for which a precomputed FFPred3 characterization of protein sequences was publicly available. Based on these considerations, the Slim *D. melanogaster* dataset was first assembled (see Methods). A first insight on the annotation power of the three methods was assessed from ROC curves. Taking into account the natural imbalance between positively and negatively protein sequences annotated to each GO-CC category and the importance of positively annotated protein sequences, PR curves were complementary analyzed. Both ROC and PR curves showed promising comparative results for the FGGA-CC^+^ method (see Fig. 4). These results were further confirmed by precision, recall and F-score evaluations. In this regard, a significant effect (p < 0.01; Friedman’s test) of annotation methods on the F-score was first observed (see Table Supplementary S3); a significant difference in favor of the FGGA-CC^+^ method (p < 0.01; Wilcoxon test with Bonferroni correction) was afterwards observed (see Table 3, left). Finally, to shed further light on the actual comparative performance FGGA-CC^+^, FFPred3 and CELLO2GO methods taking into account consistency issues, hierarchical performance metrics were considered. For this end, predicted GO-CC categories by FFPred3 and CELLO2GO methods were first propagated to parent GO-CC terms. The advantages of the FGGA-CC + method could be then clearly observed (see Table 3, right).

**Table 3:** FGGA-CC^+^, CELLO2GO, and FFPred3 methods are considered for the GO-CC annotation of protein sequences in the *Slim D. melanogaster* dataset.

Method	Precision	Recall	F-score	HP	HR	HF
FGGA-CC^+^	0.54	0.64	**0.56**	0.72	0.68	**0.68**
CELLO2GO	0.65	0.51	0.53	0.72	0.55	0.61
FFPred3	0.50	0.60	0.52	0.71	0.62	0.60

Both flat (Precision, ecall and F-score) and hierarchical (HP, HR and HF) performance metrics are considered; average results are reported. The best performing method according to the F-score or HF metrics (*p* < 0.01; Wilcoxon test with Bonferroni correction) is shown in bold.

### FGGA-CC^+^ validation with the annotation of sHSPs in *S. lycopersicum*

In plants, fruit maturation and oxidative stress can induce small Heat Shock Proteins (sHSPs) synthesis to maintain cellular homeostasis. The diversity of the sHSP gene family is mostly supported by gene duplication events that result in genetic redundancy^41^. Protein SCL plays a key role in the functional diversification process of duplicated genes as follows from the differential distribution of their proteins across different subcellular compartments^42^. Current GO-CC annotation of the sHSP gene family in *S. lycopersicum* genome remains scarce, with less than 10 in a set of 33 gene family members having some GO-CC annotation. Here, we focus on the *in-silico* GO-CC annotation of three clusters (I, II and III) of tandem duplicated sHSP genes in *S. lycopersicum*. These three clusters involve a total of nine sHSP genes, six of them without a GO-CC annotation. In addition, at each of them, one representative sHSP gene with a GO-CC annotation supported by some experimental evidence is present (Solyc06g076520, Solyc08g062450, and Solyc08g078700). In this regard, we recall that a cytosolic SCL annotation has been reported for Solyc06g076520^43^ belonging to cluster I comprising four sHSP genes in chromosome 6, that a chloroplast SCL annotation has been reported for Solyc08g062450^44^ belonging to a cluster II comprising two sHSP genes in chromosome 8, and that a mitochondria SCL annotation has been reported for Solyc08g078700^45^ belonging to cluster III comprising three sHSP genes also in chromosome 8. Aiming to shed light on the GO-CC annotation of the six remaining sHSP genes without a GO-CC annotation, a FGGA-CC^+^ classifier trained on *A. thaliana* protein sequences was considered. Owing to the tandem constraint, we further expect that FGGA-CC^+^ annotations are consistent with the common ancestral origin of sHSP genes within each cluster, i.e., cytosolic, chloroplast, and mitochondrial related GO-CC annotations are respectively expected for sHSP genes in Clusters I, II, and III. Recalling that for hierarchical ensembles of classifiers like FGGA-CC^+^, a prediction is considered correct as long as the actual solution is contained in the predicted graph, the three positive GO-CC annotation controls were verified (see Table 4 and Figures Supplementary S4–S6). Expected GO-CC annotations within the three clusters were confirmed for all but one, with Cluster I and II retaining their ancestral cytosolic and chloroplastic localization respectively. Meanwhile, in Cluster III, the mitochondrial localization was only predicted for Solyc08g078700 and Solyc08g078720. For the remaining related gene Solyc08g078710, a nonspecific organelle localization was predicted, possible due to lack of some peptide signal, adding further evidence for its gene pseudogenization process.

**Table 4:** GO-CC annotation of the *S. lycopersicum* sHSP genes with FGGA-CC^+^ classifiers.

Gene ID	DGE	Expected	Predicted GO-CC *leaf* terms
Solyc06g076520	Up	**cytosolic**	nucleoplasm, **cytosol**, chloroplast envelope, NADH dehydrogenase complex, symplast, plastid thylakoid and inner mitochondrial membrane protein complex
Solyc06g076540	Up	cytosolic	nucleoplasm, **cytosol**, chloroplast envelope, NADH dehydrogenase complex, photosynthetic membrane, mitochondrial respiratory chain and inner mitochondrial membrane protein complex
Solyc06g076560	Up	cytosolic	**cytosolic ribosome**, chloroplast envelope, NADH dehydrogenase complex, symplast, plasma membrane, nucleolus and inner mitochondrial membrane protein complex
Solyc06g076570	Up	cytosolic	nucleoplasm, **cytosolic ribosome**, chloroplast envelope, chloroplast thylakoid, photosynthetic membrane, symplast, plasma membrane and mitochondrial inner membrane
Solyc08g062450	Up	**chloroplastic**	cell-cell junction, cell periphery, **chloroplast** and nucleus
Solyc08g062340	Up	chloroplastic	cytosolic small ribosomal subunit, plasmodesma, **chloroplast**, nucleoplasm, mitochondrial membrane, nucleolus and plasma membrane
Solyc08g078700	Up	**mitochondrial**	plastid, **mitochondrial membrane**, organelle lumen and intracellular non-membrane-bounded organelle
Solyc08g078710	NDE	mitochondrial	organelle
Solyc08g078720	NE	mitochondrial	cytosolic ribosome, chloroplast thylakoid membrane, chloroplast envelope, and **mitochondrial respiratory chain complex I**

Nine tandem duplicated sHSP genes (Gene ID) are considered. Differential gene expression (DGE) profiles during fruit ripening, i.e., up-regulated (Up), not differentially expressed (NDE) or not expressed at all (NE), are included. Positive GO-CC annotation controls are shown in bold.

## Discussion and Conclusions

A graphical model-based machine learning approach for the automated and consistent GO-CC annotation of protein sequences has been presented. In this approach, a novel class of hierarchical classifiers, named FGGA-CC^+^, map the GO-CC protein annotation problem to that of discovering hidden nodes in factor graphs defined by, latent GO-CC and GO-BP categories, semantic relationships between categories, observable predictions of individual categories, and probability density functions modeling the prediction noise over individual categories. As a result, inconsistencies among observable GO-CC and GO-BP predictions -issued by binary SVM classifiers- can be transparently handled the well-known iterative Sum-Product algorithm in factor graphs. At the end of this leveraging process, a set of consistent GO-CC and GO-BP annotations are obtained. These computational modeling efforts are paid off when observing the improvement of AUC scores accomplished by native FGGA-CC classifiers with regard to naive ensembles of binary SVM classifiers. Similarly, they are pay-off when observing the improvement of AUC and hierarchical performance metrics of FGGA-CC^+^ classifiers with regard to their *native* FGGA-CC alternatives. Concerning mandatory comparisons of FGGA-CC^+^ classifiers with state of art GO-CC annotation methods like FFPred3 and CELLO2GO, flat performance metrics were first considered. In the former case, significant improvements on F-score results were observed. However, in terms of precision performance, the CELLO2GO method performed better, but at lower recall levels. We wonder if we could reach CELLO2GO precision levels (0.65) by raising the threshold of our decisions. In effect, we found that by raising the decision threshold from 0.5 to 0.9, an average precision of 0.65 with an average recall of 0.57 (F-score of 0.56) was accomplished (see Supplementary Table S4), suggesting that FGGA-CC^+^ classifiers can accommodate a wide spectrum of precision/recall requirements. To shed light on the actual comparative performance of the FGGA-CC^+^, FFPred3 and CELLO2GO methods, hierarchical performance metrics were then considered. In these evaluations, the advantages of the FGGA-CC^+^ method could be clearly observed, with comparable results in terms of the hierarchical precision and significant better results in terms of the hierarchical recall.

For purposes of FGGA-CC^+^ validation, the challenging annotation of nine sHSP genes of tandem duplication origin in the tomato genome was considered. Verification of three positive controls allowed us to tackle the *in-silico* annotation of the six remaining sHSP genes. Consistent GO-CC annotation results were mostly observed with the sole exception of Solyc08g078710, for which a nonspecific organelle prediction, instead of a mitochondrial one, was obtained. Posterior analysis of the sHSP characterization patterns used for FGGA-CC^+^ queries revealed that differently from its two tandem duplicated counterparts, Solyc08g078710 lacks of a key signal allowing its transport into the mitochondria. This signal is the Tom20 motif, a mitochondrial targeting signal expected at the N-terminal presequences that is recognized by the Tom20 import receptor at the outer mitochondrial membrane^46^. This finding points out the importance of using a comprehensive, GO-CC specific, characterization of protein sequences for their reliable GO-CC annotation.

Computational and biological concepts underlying the design of FGGA-CC^+^ classifiers, from techniques used for the characterization of protein sequences, to the factor graph modeling of target GO-CC subgraphs, including the integration of GO-BP knowledge and the modeling of GO-CC prediction noise at flat binary SVM classifiers, provide a systematic framework for designing a computational tool allowing the integral GO annotation of protein sequences. By characterizing a sufficient large collection of annotated proteins in the three GO subdomains, including those coming from orthologous protein coding genes, it should be possible to provide accurate and integral and precise GO annotations of protein coding genes in many non model organisms. Finally, as pointed out in^47^, and demonstrated in a series of recent publications^33,48,49^, user-friendly and publicly accessible web-servers represent the future direction for developing practically more useful prediction methods and computational tools. In this regard, we shall make efforts in our future work to provide a web-server for the method presented in this paper.

## Methods

To develop really useful sequence-based statistical classifiers for a biological system, such as those reported in a series of recent publications^33,48,50^, one should observe the 5-step rule^51^. As a result, one should make the following five steps very clear: (*i*) how to construct a valid benchmark dataset to train and test the classifiers; (*ii*) how to characterize protein sequences so that they can reflect their intrinsic correlation with target categories; (*iii*) how to develop a powerful algorithm for predicting target categories; (*iv*) how to properly perform cross-validation tests to objectively evaluate the anticipated accuracy of classifiers; and (*v*) how to establish a user-friendly web-server for the classifiers that is accessible to the public. In what follows, we describe how to deal with these steps one-by-one for the specific case of FGGA-CC^+^ classifiers and the prediction of GO-CC categories.

### Datasets

#### Benchmark datasets

GO-CC annotation data with experimental and computational evidence codes [http://geneontology.org/page/guide-go-evidence-codes] was first collected. Regarding experimental codes, Inferred from Experiment (EXP), inferred from Direct Assay (IDA), Inferred from Physical Interaction (IPI), Inferred from Mutant Phenotype (IMP), inferred from Genetic Interaction (IGI) and Inferred from Expression Pattern (IEP), were considered. Regarding computational evidence codes, inferred from Sequence or structural Similarity (ISS), inferred from Sequence Orthology (ISO), inferred from Sequence Alignment (ISA) and inferred from Sequence Model (ISM), were considered. In addition, annotation data was also collected for GO-BP boundary nodes, i.e., GO-BP terms connected to GO-CC terms through the non-transitive *occurs_in* relationship. For GO-BP boundary nodes, we considered annotation data with experimental, computational or Inferred from Electronic Annotation (IEA) evidence codes. This kind of *soft* annotation data policy for GO-BP boundary nodes aims to compensate the lack of sufficient protein sequences with experimental or computational evidence codes that may overshadow the actual power of GO-BP boundary nodes for the enhancement of GO-CC predictions. Regarding minimum requirements for learning individual GO terms with binary SVM classifiers, a minimum of 50 positively annotated protein sequences was considered. In addition, to assemble conveniently balanced binary training datasets^52^, positively annotated protein sequences were complemented with negative annotated protein counterparts using the *inclusive* separation policy^53^. As shown in Table 5, datasets comprising both positively and negatively GO-CC annotated protein sequences from five models organisms, *D. rario*^54^, *A. thaliana*^55^, *S. cerevisiae*^56^, *D. melanogaster*^57^ and *M. musculus*^58^, were finally assembled.

**Table 5:** Annotation datasets used for the prediction of GO-CC categories.

Organism	# GO-CC terms	# *Soft* GO-BP terms	# Samples
*A. thaliana*	143	8	22778
*M. musculus*	304	17	13417
*D. melanogaster*	167	11	6176
*S. cerevisiae*	174	12	5134
*D. rario*	52	1	1243

Protein sequences from five model organisms are considered. The number of GO-CC terms, with the number of *soft* GO-BP boundary terms used for the enhancement GO-CC predictions along with the number of annotated samples, are shown.

#### Slim D. melanogaster dataset

*D. melanogaster* protein sequences were collated from the UniProt database based on their annotation to any of the 89 GO-CC categories predefined by the FFPred3 method; to allow fair hierarchical comparisons of GO-CC predictions between methods, 22 ancestor GO-CC categories were also included. In addition, the same evidence codes of benchmark datasets were used. Taking into account the high computational overhead of FFPred3 GO-CC predictions, a reduced set of 270 protein sequences was finally considered (see Supplementary Data file 1).

#### *S. lycopersicum* (cv. Heinz 1706) sHSP dataset

Although the tomato reference genome was published in 2012, the functionality of sHSP genes in this model organism for fleshy fruit development remains mostly unknown. Using a transcriptomic (RNA-seq) and evolutionary genomic approach, a family of thirty-three sHSP genes in *S. lycopersicum* (cv. Heinz 1706) genome was recently established^41^. Here, the GO-CC annotation of this gene family is considered. We restrict our attention to tandem duplicated sHSP genes arranged into physical clusters with at least one of its members being up-regulated during fruit ripening to ensure functionality, and with a established SCL annotation to validate annotations at each cluster. As a result, the GO-CC annotation of nine tandem duplicated genes sHSP genes arranged into three physical clusters was tackled: a cluster of four members (Solyc06g076520, Solyc06g076540, Solyc06g076560, Solyc06g076570) in chromosome 6, a cluster of two members (Solyc08g078710, Solyc08g078720) in chromosome 8, and another cluster of three members (Solyc08g062340, Solyc08g062450, Solyc08g078700) also in chromosome 8.

### Protein sequence representation

Protein sequences were characterized in terms of a fixed number features. As shown in Table 6, four characterization methods were analyzed: (i) Signal^+^, encoding features used by well-known TargetP^27^, SignalP^59^, Transmembrane Helices^60^, WoLF PSORT^61^, and MitoFates^62^ tools for the prediction of standard SCL categories, along with the presence/absence of localization signals collected in the LocSigDB^63^ database, (ii) PrositeBin encoding just the presence/absence of Prosite domains, (iii) Signal^+ +^, encoding features in the Signal^+^ and PrositeBin characterizations, and (iv) Physicochemical^+^, encoding features in the Signal^+^ characterization, together with of physicochemical and secondary structure properties^64–67^. Characterization methods were implemented with in-house R scripts. To assess the effect of characterization methods in the prediction power of FGGA-CC^+^ classifiers, the largest annotation dataset (*A. thaliana*) was considered.

**Table 6:** Characterization methods for protein sequences.

Method	Features	# Features
Signal^+^	Established predictors of standard SCLcategories + LocSigDB signals	96
PrositeBin	Presence/absence of Prosite domains	1354
Signal^+ +^	Signal^+^ + PrositeBin	1450
Physicochemical^+^	Signal^+^ + Physicochemical and secondary structure properties	165

### Consistent GO-CC annotation of protein sequences with FGGA-CC^+^ classifiers

**Figure 5:**
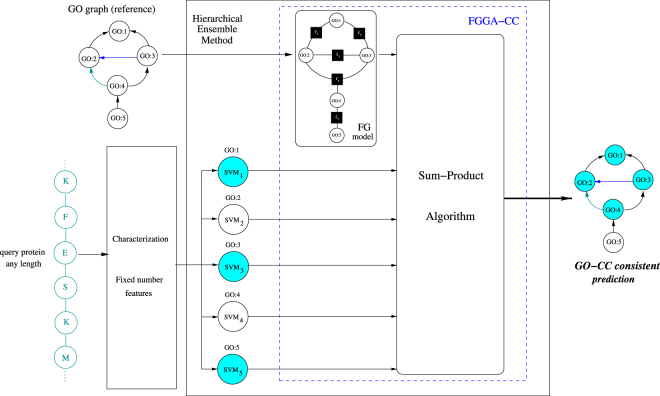
GO-CC annotation of a protein sequences with FGGA-CC classifiers. A GO-CC subgraph defining the expected structure of GO-CC predictions is first converted to a factor graph (FG) model. Protein sequences of any length are characterized in terms of a fixed number of features. Flat binary SVM classifiers (*SVM*_*i*_) predict individual GO-CC categories (GO:i) upon protein sequence queries. Flat, likely inconsistent, binary GO-CC predictions are leveraged by executing the Sum-Product algorithm on the FG model. At the end, a set of consistent GO-CC predictions is obtained.

GO-CC annotation of protein sequences was first tackled with *native* FGGA-CC classifiers, hierarchical ensembles of binary SVM classifiers relying on the power of factor graph models for overcoming inconsistencies among flat SVM predictions of individual GO-CC categories (see Fig. 5). FGGA-CC classifiers arise as a natural extension of FGAA classifiers originally developed for the automated and consistent GO-MF annotation of protein coding genes. Both the GO-MF and GO-CC subontologies make extensive use of the fundamental transitive *is-a* relationship. In addition, the GO-CC subontology makes extensive use of the transitive *part of* relationship. Both relationships are considered by *native* FGGA-CC classifiers when performing GO-CC annotations. Aiming to accomplish more accurate GO-CC annotations, the integration of SCL knowledge from GO-BP boundary terms was additionally considered. As a result, *native* FGGA-CC classifiers were further extended into FGGA-CC^+^ classifiers. To accomplish this extension, the non-transitive *occurs-in* relationship between GO-CC and GO-BP terms was further considered. However, since non-transitive relationships may lead to non-transitive inference paths precluding the free propagation^68,69^ and consistency checking of GO-CC annotations in supporting factor graphs, a transitive closure screening process was introduced prior to factor graph modeling. After transitive closure processing, the resulting GO subgraph is ready to be transformed into a factor graph classification model using roughly the same methodology described in^40^.

#### Transitive Closure Screening of GO-CC subgraphs enriched with GO-BP boundary nodes

Given a GO-CC subgraph enriched with GO-BP boundary nodes, a transitive closure screening process is performed using a Depth-First Search (DFS) algorithm^70^ ignoring repeated nodes. Starting from a bottom-leaf node, a link between a child node and its parent node is accepted only if, for *all* grandparent nodes, the boolean function *h* is satisfied for all composite child-parent-grandparent relationships. If any of these *h* evaluations fail, the child-parent link is deleted. In the definition of *h*, all reasoning rules established for standard^71^ and experimental^72^ relationships, like the *occurs_in*, are considered (see Table 7).

**Table 7:** Transitive closure screening of a GO subgraph by means of a boolean function *h*.

*GO*:*j* → *GO*:*j* → *GO*:*z*		*h*	*GO*:*i* → ;*GO*:*j* → *GO*:*z*		*h*
is a	is a	1	regulates	is a	1
is a	part of	1	regulates	part of	1
is a	regulates	1	regulates	regulates	0
is a	occurs in	1	regulates	occurs in	0
part of	is a	1	occurs in	is a	1
part of	part of	1	occurs in	part of	1
part of	regulates	0	occurs in	regulates	0
part of	occurs in	1	occurs in	occurs in	0

The admissibility of composite relationships between a GO term *GO:i*, its parent *GO:j*, and its grandparent *GO:z*, are checked by *h*.

**Figure 6:**
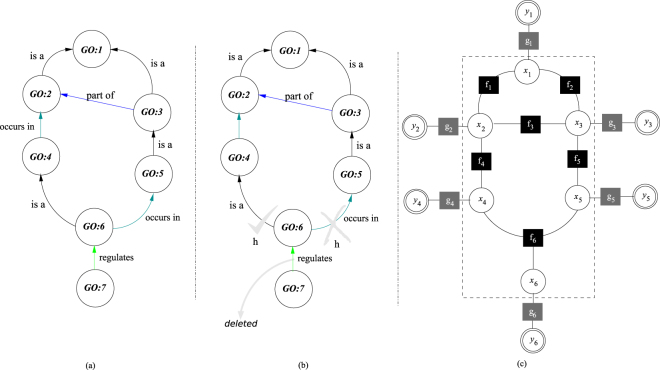
(**a**) Original GO subgraph (**b**) After checking the graph transitive closure with the boolean function *h*, the link *GO:7* → *GO:6* is deleted (**c**) Factor graph model used for GO-CC predictions. Inside the dash lined box, a core factor graph contains binary variable nodes *x*_*i*_ modeling GO terms, and boolean factor nodes *f*_*j*_ modeling relationships between them. Outside the dash lined box, the core factor graph is enriched with observable, real-valued, variable nodes *y*_*i*_ modeling independent GO-CC predictions, and probabilistic factor nodes *g*_*i*_ modeling corresponding prediction noise.

For example, in Fig. 6a, the transitive closure of inference paths in a GO subgraph including non-transitive relationships is analyzed. In particular, the presence of the *GO:7* → *GO:6* link involving the non-transitive *regulates* relationship is evaluated. Since *h* is verified by composite path *GO:7* → *GO:6* → *GO:4* but is rejected by composite path *GO:7* → *GO:6* → *GO:5*, the *GO:7* → *GO:6* link gets removed (see Fig. 6b).

#### Factor graph transformation and inference of GO-CC annotations

After transitive closure screening, the resulting GO subgraph is first transformed into a core factor graph (see Fig. 6c). For this purpose, GO terms are mapped to binary variable nodes and relationships between GO terms are mapped to logical factor nodes -logical functions- implementing the True Path Graph (TPG) constraint. Specifically, the TPG constraint ensures that if a child GO term is annotated positive, then its parent GO term(s) must also be annotated positive; on the other hand, if a parent GO term is annotated negative, then its children GO term(s) must also be annotated negative. The core factor graph is then enriched with observable variable nodes and probabilistic factor nodes. Observed variable nodes model practical binary SVM predictions over ideal, but hidden/latent, variable nodes in the core factor graph. On the other hand, probabilistic factor nodes model zero mean Gaussian distributions modeling the prediction noise of practical SVM classifiers.

For a given query protein sequence, GO-CC annotations are obtained by the execution of the iterative Sum-Product algorithm between nodes of the enriched factor graph. The algorithm starts from the observable but noisy predictions at leaf nodes of the factor graph. After a few number of iterations, approximated APPs on hidden variable nodes -target GO-CC categories- can be obtained^40^. We recall that only approximated APPs can be guaranteed since cycles^73^ are naturally expected in GO-CC compliant factor graphs. From these probabilities, corresponding MAP estimates -minimizing the probability of erroneous GO-CC predictions- are obtained; practically, a maximum of 50 iterations were allowed. Note that since our GO-CC predictions follow from MAP estimates, we do not expect they are able to optimize more elaborate performance metrics like the F-score. We note, however, that the design of optimal F-score classification algorithms remain a challenging computational problem even for the prediction of flat multiclass/multilabel categories^74^.

Soft-margin SVM classifiers with a radial basis function kernel and default parameters were used for the prediction of individual GO-CC and GO-BP categories. To fulfill the assumption of zero-mean Gaussian prediction noise, the margins of SVM classifier outputs were used. A complementary validation stage after the training of SVM classifiers was used to assess the standard deviation of Gaussian distributions modeling the prediction noise of individual GO-CC and GO-BP categories. Practically, SVMs classifiers were implemented with e-1071 R package^75^. In addition, the factor graph iterative Sum-Product algorithm was implemented with in-house R [https://cran.r-project.org/] scripts.

### Performance evaluation

The prediction performance of *native* FGGA-CC and FGGA-CC^+^ classifiers was evaluated with 5-fold cross-validation tests. Taking into account the hierarchical relations among target GO-CC categories, both flat hierarchical classification performance metrics were considered^76^. Differently from their flat counterparts, hierarchical classification performance metrics appropriately recognize partially correct classifications and correspondingly penalize more distant or more superficial errors -prediction errors at upper levels of a hierarchy should be punished more severely that those at deeper levels^77^. In particular, the hierarchical precision (HP), the hierarchical recall (HR), and the hierarchical F-score (HF) measures introduced in^78^ were used. Below are their formulas:

1 $$HP(s)=\frac{1}{|l({P}_{G}(s))|}\,\sum _{q\,\in \,l({P}_{G}(s))}\,\mathop{{\rm{\max }}}\limits_{c\,\in \,l({C}_{G}(s))}\frac{|\,\uparrow c\cap \uparrow q|}{\uparrow q}$$

2 $$HR(s)=\frac{1}{|l({C}_{G}(s))|}\,\sum _{c\,\in \,({C}_{G}(s))}\,\mathop{{\rm{\max }}}\limits_{q\,\in \,l({P}_{G}(s))}\frac{|\,\uparrow c\cap \uparrow q|}{\uparrow c}$$

3 $$HF(s)=\frac{2\cdot HP\cdot HR}{HP+HR}$$

where *s* is a protein sequence, *G* is GO subgraph, *P*_*G*_(*s*) ⊂ *G* is the predicted GO subgraph of *s*, *C*_*G*_(*s*) ⊂ *G* is the actual GO subgraph of *s*, *l*(*P*_*G*_(*s*)) is the set of leaves of the *P*_*G*_(*s*) and *l*(*C*_*G*_(*s*)) is the set of leaves of *C*_*G*_(*s*). In addition, ↑*q* is the set of ancestors of a node *q* belonging to *P*_*G*_(*s*), and ↑*c* is the set of ancestors of a node *c* belonging to *C*_*GO*_(*s*). Concerning fair comparisons of FGGA-CC^+^ classifiers against established, but not hierarchical, methods for the automated GO-CC annotation of protein coding genes, the average precision, the average recall and the average F-score performance metrics were used. Specifically, for each protein sequence *s*, the precision *p*(*s*) was calculated as $\frac{tp(s)}{tp(s)+fp(s)}$, the recall *r*(*s*) as $\frac{tp(s)}{tp(s)+fn(s)}$ and the F-score as $\frac{2\cdot p(s)\cdot r(s)}{p(s)+r(s)}$, where *tp* is the number of GO-CC categories correctly predicted as positives (true positives), *fp* is the number of GO-CC categories incorrectly predicted as positives (false positives) and *fn* is the number of GO-CC categories incorrectly predicted as negatives (false negatives).

### Evaluation protocol

Firstly, the annotation performance of *native* FGGA-CC classifiers was evaluated against that of flat ensembles of binary SVM classifiers. Secondly, the annotation performance of (GO-BP enriched) FGGA-CC^+^ classifiers was evaluated against that of *native* FGGA-CC counterparts. In the former case, average AUC scores^79^ at individual GO-CC categories were additionally computed using the facilities in the PerfMeas package^80^. FGGA-CC^+^ classifiers were also evaluated against two established methods for GO-CC annotation, FFPred3 and CELLO2GO; literature results on these methods have been reported only with flat performance metrics. Aiming fair comparisons, both flat and hierarchical performance metrics were used. For these evaluations, the *Slim D. melanogaster* dataset was used. In all cases, the statistical significance of observed differences was assessed with the Friedman and Wilcoxon rank sum tests at *α* = 0.01 significance level.

## Electronic supplementary material

Supplementary Information

Dataset 1
