# Supplementary material for: Consistent prediction of GO protein localization
Source: Sci Rep. 2018 May 17;8:7757. doi: 10.1038/s41598-018-26041-z (PMC5958134; doi:10.1038/s41598-018-26041-z)
Supplement: Supplementary file 1 — Supplementary Information [file 41598_2018_26041_MOESM1_ESM.pdf]

# Supplementary Information
# Consistent prediction of GO protein localization

Flavio E. Spetale, Debora Arce, Flavia Krsticevic,
Pilar Bulacio and Elizabeth Tapia

March 12, 2018

Table S 1: Characterization methods on the *A. thaliana* dataset. Friedman's test results.

| Method | Mean Rank |
|---|---|
| $Prosite_b$ | 2.259 |
| $SS^+$ | 2.105 |
| $SS^{++}$ | 1.984 |
| $Physicochemical^+$ | 1.711 |

Table S 2: Characterization methods on the *A. thaliana* dataset. Pairwise comparisons using the Wilcoxon rank sum test and $p_{value}$ with Bonferroni correction.

| | $Physicochemical^+$ | $SS^{++}$ | $SS^+$ |
|---|---|---|---|
| $SS^{++}$ | 0.00629 | - | - |
| $SS^+$ | 0.00019 | 0.02221 | - |
| $Prosite_b$ | 0.00142 | 0.00624 | 0.42320 |

Table S 3: Pairwise comparisons of GO-CC annotation method on the *Slim D. melanogaster* dataset. Wilcoxon rank sum test and $p_{value}$ with Bonferroni correction.

| | FGGA-$CC^+$ | CELLO2GO |
|---|---|---|
| CELLO2GO | $< 2x10^{-16}$ | - |
| FFPred3 | 0.0025 | $< 2x10^{-16}$ |

Table S 4: The average precision, recall and F-score perfomance of FGGA-$CC^+$ classifiers on the *Slim D. melanogaster* dataset for increasing decision threshold values.

| threshold | Precision | Recall | F-score |
|---|---|---|---|
| 0.6 | 0.58 | 0.62 | 0.56 |
| 0.7 | 0.60 | 0.61 | 0.56 |
| 0.8 | 0.62 | 0.59 | 0.56 |
| 0.9 | 0.65 | 0.57 | 0.56 |

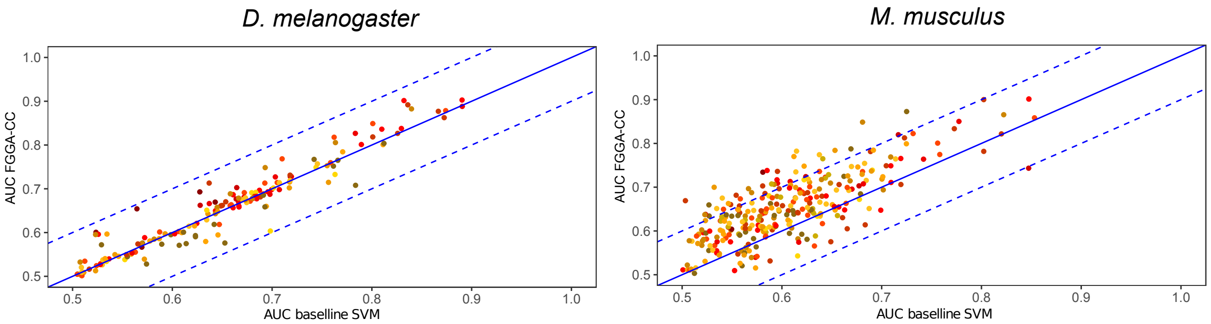


Figure S 1: Scatter plots of the average AUC scores attained by FGGA-CC and baseline ensembles of SVM classifiers when performing the GO-CC annotation of protein sequences characterized by the Physicochemical$^+$ method. As deeper GO-CC categories are considered, points in the scatter plot turn from yellow to red.

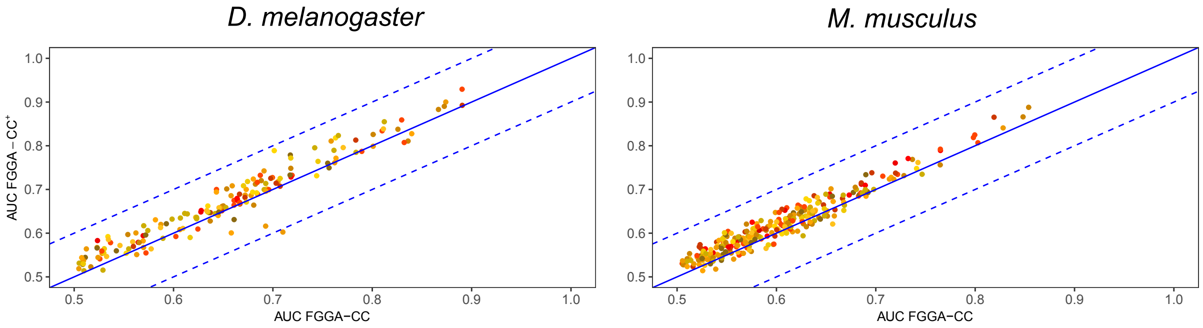


Figure S 2: Scatter plots of the average AUC scores attained by FGGA-CC$^+$ and *native* FGGA-CC when performing the GO-CC annotation of protein sequences characterized by the Physicochemical$^+$ method. As deeper GO-CC categories are considered, points in the scatter plot turn from yellow to red.

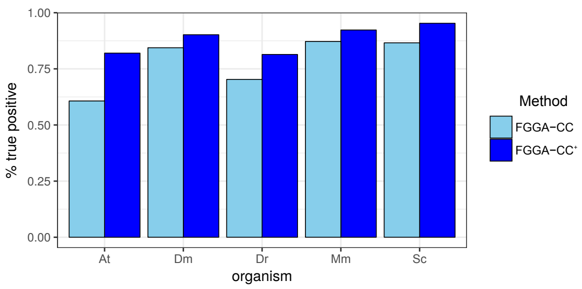


Figure S 3: Bar graph showing the normalized percentage of true positives using FGGA-CC and FGGA-CC$^+$ for five model organisms.

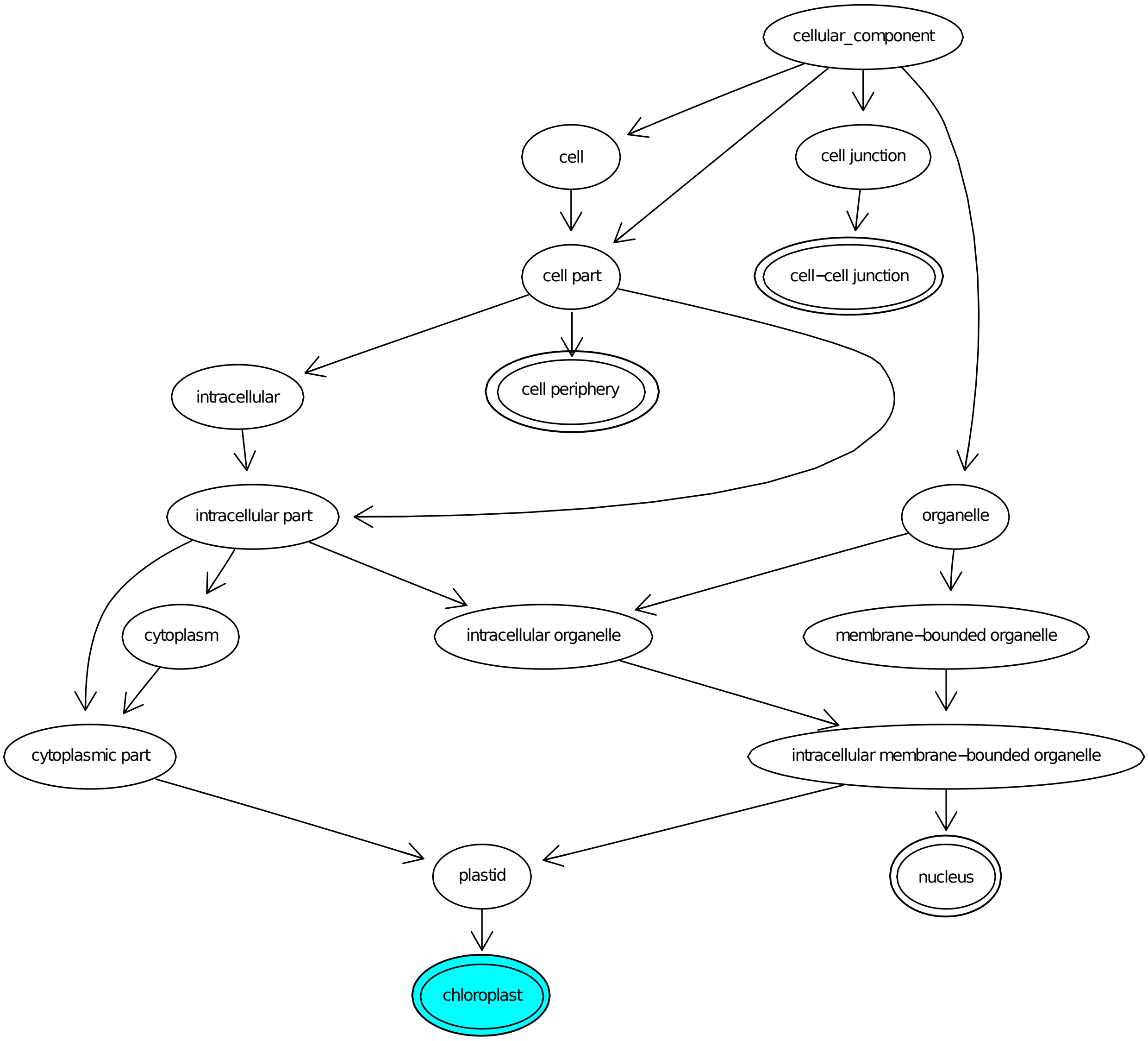


Figure S 4: **Positive control Solyc08g062450.** Predicted graph for Solyc08g062450. A FGGA-CC$^+$ classifier trained with *A. thaliana* annotation pretein data (Physicochemical$^+$ characterization) is used. The predicted graph contains 17 GO-CC terms (4 leaves - double circle); the experimentally reported GO-CC annotation (chloroplast - sky blue) is included as a leaf.

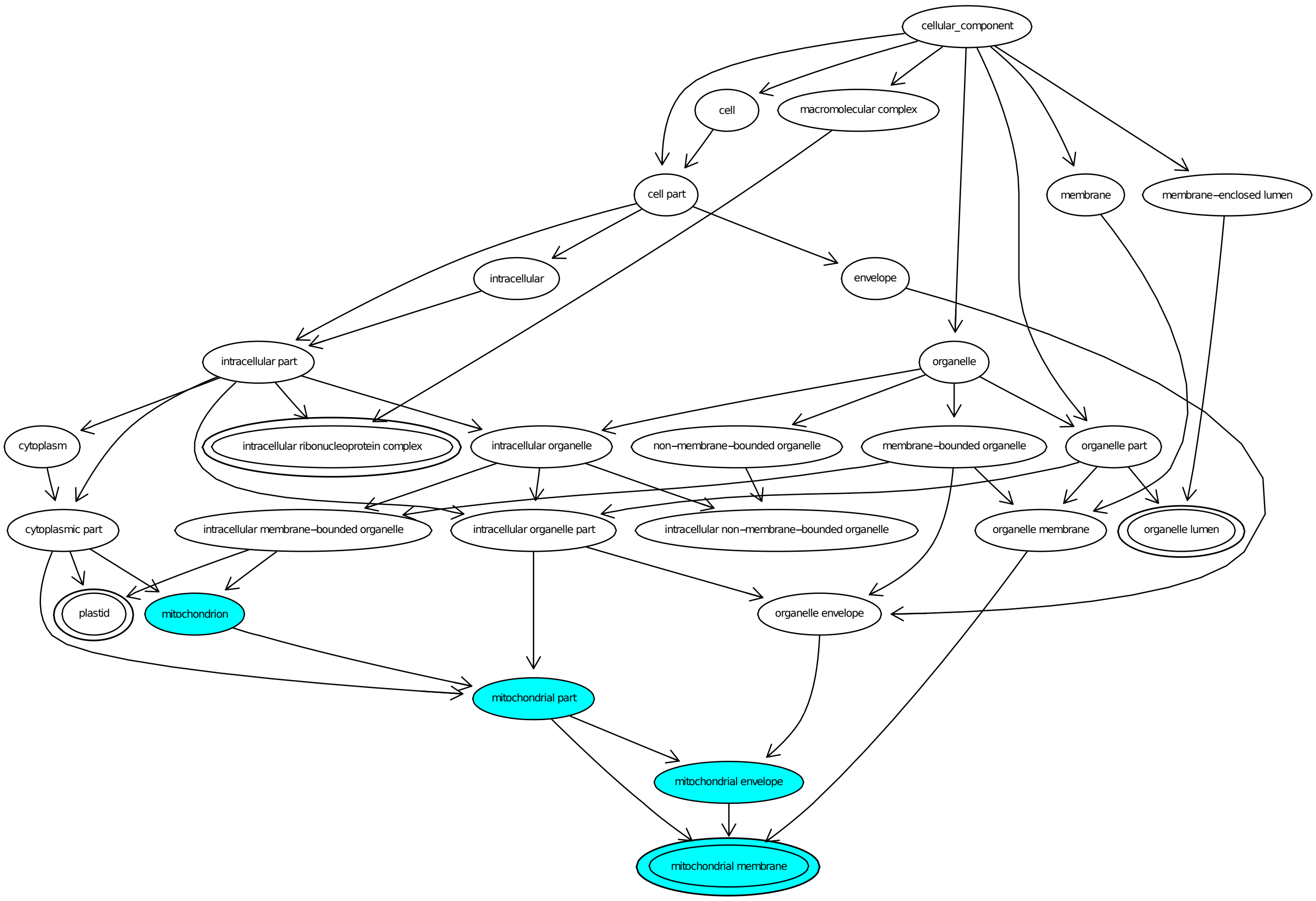


Figure S 5: **Positive control Solyc08g078700.** Predicted graph for Solyc08g078700. A FGGA-CC$^{+}$ classifier trained with *A. thaliana* annotation protein data (Physicochemical$^{+}$ characterization) is used. The predicted graph contains 29 GO-CC terms (4 leaves - double circle); the experimentally reported GO-CC annotation (mitochondrion - sky blue) is included as an ancestor of a more specific leaf prediction (mitochondrial membrame).

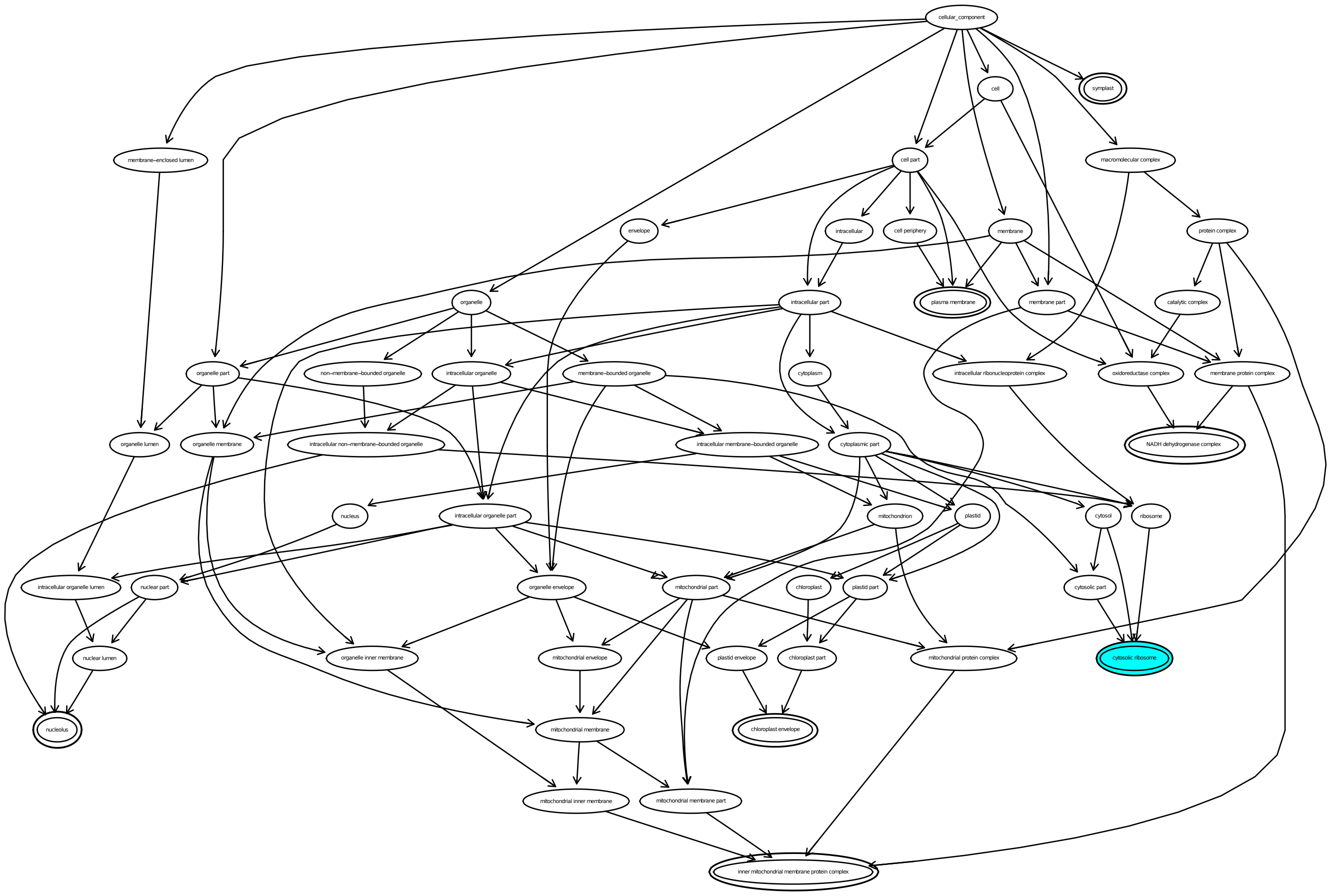


Figure S 6: **Solyc06g076560 of unknown subcellular localization.** Predicted graph for Solyc06g076560. A FGGA-CC$^+$ classifier trained with *A. thaliana* annotation protein data (Physicochemical$^+$ characterization) is used. The predicted graph contains 58 GO-CC terms (7 leaves - double circle); the experimentally reported GO-CC annotation (cytosolic ribosome - sky blue) is included as a leaf.
